# Intracellular localization and interaction of mRNA binding proteins as detected by FRET

**DOI:** 10.1186/1471-2121-11-69

**Published:** 2010-09-15

**Authors:** Pamela S David Gerecht, Molly A Taylor, J David Port

**Affiliations:** 1 Departments of Medicine/Cardiology and Pharmacology, University of Colorado School of Medicine, 12700 East 19th Avenue, Aurora, CO 80045, USA; 2 Department of Pharmacology, Case Western Reserve University, 2103 Cornell Road, Cleveland, OH 44106, USA; 3 University of Colorado School of Medicine Division of Cardiology, B139 12700 East 19th Avenue, Rm 8001 Aurora, CO 80045, USA

## Abstract

**Background:**

A number of RNA binding proteins (BPs) bind to A+U rich elements (AREs), commonly present within 3'UTRs of highly regulated RNAs. Individual RNA-BPs proteins can modulate RNA stability, RNA localization, and/or translational efficiency. Although biochemical studies have demonstrated selectivity of ARE-BPs for individual RNAs, less certain is the *in vivo *composition of RNA-BP multiprotein complexes and how their composition is affected by signaling events and intracellular localization. Using FRET, we previously demonstrated that two ARE-BPs, HuR and AUF1, form stable homomeric and heteromeric associations in the nucleus and cytoplasm. In the current study, we use immuno-FRET of endogenous proteins to examine the intracellular localization and interactions of HuR and AUF1 as well as KSRP, TIA-1, and Hedls. These results were compared to those obtained with their exogenously expressed, fluorescently labeled counterparts.

**Results:**

All ARE-BPs examined were found to colocalize and to form stable associations with selected other RNA-BPs in one or more cellular locations variably including the nucleus, cytoplasm (in general), or in stress granules or P bodies. Interestingly, FRET based interaction of the translational suppressor, TIA-1, and the decapping protein, Hedls, was found to occur at the interface of stress granules and P bodies, dynamic sites of intracellular RNA storage and/or turnover. To explore the physical interactions of RNA-BPs with ARE containing RNAs, *in vitro *transcribed Cy3-labeled RNA was transfected into cells. Interestingly, Cy3-RNA was found to coalesce in P body like punctate structures and, by FRET, was found to interact with the RNA decapping proteins, Hedls and Dcp1.

**Conclusions:**

Biochemical methodologies, such as co-immunoprecipitation, and cell biological approaches such as standard confocal microscopy are useful in demonstrating the possibility of proteins and/or proteins and RNAs interacting. However, as demonstrated herein, colocalization of proteins and proteins and RNA is not always indicative of interaction. To this point, using FRET and immuno-FRET, we have demonstrated that RNA-BPs can visually colocalize without producing a FRET signal. In contrast, proteins that appear to be delimited to one or another intracellular compartment can be shown to interact when those compartments are juxtaposed.

## Background

RNA binding proteins (BPs) are involved in numerous aspects of RNA maturation, turnover, translational efficiency, and in movement of transcripts throughout the cell. RNA BPs have roles during normal cellular growth and division, as well as during times of cellular stress, conditions that may alter the "normal" status of protein/protein and protein/mRNA interactions. Our studies focus on a class of RNA binding proteins known as ARE binding proteins. By recognizing and binding to A+U rich elements (AREs) in the 3'UTR of RNA transcripts the ARE-BPs control the cellular half life of numerous gene classes of RNA including, proto oncogenes, cytokines, chemokines, and G protein coupled receptors [1].

Several ARE-BPs have been studied extensively including: AUF1/hnRNP D [2], BRF1/2 [3], HuR/ELAV [4], KSRP (FBP2/ZBP1/2) [5], TIA-1/TIAR [6], and TTP (ZFP36) [7,8]. HuR has been shown to exert a stabilizing effect on RNA turnover whereas TTP, KSRP, and BRF1 have all been shown to have a destabilizing role. In contrast, TIA-1 appears to facilitate translational arrest. AUF1 is unique in that it is expressed as four splice variants with each splice form having varying roles in stabilizing and destabilizing ARE containing mRNAs [9].

Coimmunoprecipitation experiments have demonstrated that various ARE-BPs can directly interact with one another including: HuR/AUF1 [10]; HuR/TIA-1 [11,12]; KSRP/AUF1 [13] and KSRP/TIA-1 [14]. What cannot be determined by immunoprecipitation, however, is where within the cell these interactions are taking place. Additional biochemical characterization has also demonstrated that more than one ARE-BP can bind to the same ARE in an additive or competitive manner: HuR/AUF1 [15-17]; HuR/TIA-1/KSRP [18]; HuR/KSRP [19,20]; KSRP/AUF1 [21]; TIA-1/AUF1/HuR [22,23]; and TIA-1/Hu [24].

The regulation of ARE containing RNAs extends well beyond simple interactions between various ARE-BPs. ARE-BPs are themselves subject to regulation by phosphorylation, an effect that has been shown to drive their intracellular localization and/or to alter their ability to bind to target RNAs [25-30].

Here we expand upon previous studies focusing on AUF1 and HuR to include the ARE-BPs KSRP and TIA-1. Beyond simply expanding the number of proteins, we wanted to investigate endogenously expressed proteins and their interactions and compare this to results obtained with fluorescently tagged proteins. To achieve this goal, we used the technique of immuno-FRET. This previously described but minimally utilized technique brings together immunocytochemistry and FRET to visualize endogenous proteins and their interactions in individual cells [31].

Using immunocytochemistry, we demonstrate that endogenous HuR, AUF1, KSRP and TIA-1 all colocalize in the nucleus of DDT1-MF2 cells with variable expression of each in the cytoplasm. We also show via immuno-FRET the physical interactions of all pair-wise combinations of these proteins. To extend these findings, we examined the intracellular localization and interactions of these ARE-BPs under conditions of cellular stress (induction of stress granules (SGs)) and MAP Kinase stimulation (induction of nucleocytoplasmic shuttling). Upon oxidative stress, HuR, AUF1, KSRP and TIA-1 were all found to localize to SGs. MAPK stimulation, known to induce nucleocytoplasmic shuttling of HuR and AUF1 [15], also caused movement of KSRP to the cytoplasm but had no affect on TIA-1 localization. Immuno-FRET based methods demonstrated the preservation of pair-wise ARE-BP interactions when protein pairs were present and colocalized within the cytoplasm.

We also investigated Processing bodies, (P bodies, PBs), cytoplasmic compartments associated with RNA degradation, for the presence of each ARE-BP [32]. Certain ARE-BPs, known to populate PBs have also been shown to occupy SGs, with the possibility that there is dynamic exchange of proteins between PBs and SGs. In the current studies, we used Hedls [33] and Dcp1a [34] as markers for PBs to visualize whether other ARE-BPs populate PBs and if in fact they interact when localized to PBs. In these experiments, PB formation was unaffected by MAP Kinase stimulation and cellular stress. TIA-1 was shown to associate with PBs under conditions of MAPK stimulation and cellular stress when TIA-1 was present in the cytoplasm and SGs, respectively. Lastly, we found that *in vitro *transcribed, fluorescently labeled, stabilized mRNA transcripts coding for the β adrenergic receptor are present in the cytoplasm of DDT1-MF2 cells as punctate bodies. These transcripts were shown to recruit the mRNA degradation enzymes Hedls and Dcp1a to their cellular location.

In summary, we conclude that a number of ARE-BPs are capable of interacting with one another; each protein is subject to differential regulation in terms of rate and degree of relocalization from nucleus to cytoplasm. Further distinctions arise at the level of discrete intracellular compartments, i.e., SGs and PBs. In this context, we demonstrate the utility of the immuno-FRET method to visualize the interaction between endogenous ARE-BP in a number of discrete cellular locations. Using this method we have shown that all of the four ARE-BP investigated interact in pairs in the cell within specific intracellular compartments. Moreover, by using immuno-FRET, we were able to differentiate between mere colocalization of the endogenous proteins and specific, FRET-detectable, interactions. Importantly, immuno-FRET results recapitulate substantially those obtained with fluorescently labeled proteins.

## Results

FRET is a powerful technique capable of identifying interactions between pairs of fluorescently labeled proteins with appropriately overlapping excitation and emission spectra. In this study we used both 'traditional' and immuno-FRET methods to examine the interactions of several ARE-BPs in both live and fixed cells. For traditional FRET, eCFP and eYFP tagged versions of proteins were used. For immuno-FRET, endogenously expressed proteins were detected via 2° Abs labeled with Cy3 and Cy5 (or equivalent). As with traditional FRET, immuno-FRET requires that the fluorescently labeled antibodies be FRET compatible pairs. Traditional and immuno-FRET each have advantages and disadvantages. With traditional FRET, expression levels of transiently transfected tagged proteins can be difficult to control. Results have the potential to be erroneous due to protein overexpression, which can lead to aggregation and mis localization, something that can be caused by the presence of the fluorescent protein tag itself [35]. With immuno-FRET, false-negative data could be due to i) greater distance and flexibility between fluorophors on the 2° Ab, ii) the potential for 1° or 2° antibodies epitopes to be masked in protein complexes. False-positive immuno-FRET signals could be due to i) greater distance and flexibility between fluorophors on the 2° Ab, ii) close packing of proteins in cellular compartments. However, the great advantage of immuno-FRET is the ability to detect endogenous, unmodified protein at the individual cell level. Control experiments conducted for both FRET methods are described in Materials and Methods.

### Presence and localization of ARE-BPs in DDT1-MF2 cells

**Figure 1:**
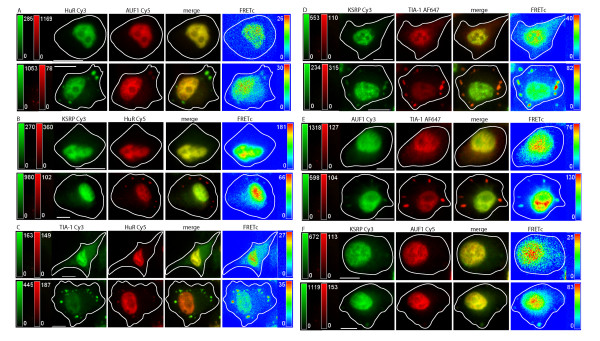
**Localization and FRET between endogenous ARE binding proteins upon oxidative stress**. ARE-BPs were detected via immunocytochemistry. Cells were treated with 0.5 mM sodium arsenite (SA) for 30 minutes. ARE-BPs are shown in control cells (top row), and SA treated cells, (bottom row) in each panel of images. Images in each row, from left to right, show the ARE-BP detected via: i) Cy3 labeled 2° Ab in green, ii) Cy5 or AF647 labeled 2° Ab in red, iii) Cy3-Cy5 merged image, iv) FRETc signal in thermal pseudocolor. Color matched signal intensity scales are indicated for each image. All scale bars are 10 μm in length. **Panel A: **HuR (Cy3) and AUF1 (Cy5). The Cy5 image intensity (bottom row) was increased to allow easier detection of AUF1 in SGs. **Panel B: **KSRP (Cy3) and HuR (Cy5). The gamma setting of the FRETc image (bottom row) was decreased to 0.81 to allow increased visualization of FRET interaction in SGs. **Panel C: **TIA-1 (Cy3) and HuR (Cy5). **Panel D: **KSRP (Cy3) and TIA-1 (AF647). **Panel E: **AUF1 (Cy3) and TIA-1 (AF647). **Panel F: **KSRP (Cy3) and AUF1 (Cy5). The gamma setting of the Cy5 image (bottom row) was decreased to 0.79 to allow increased visualization of AUF1 in SGs.

**Figure 2:**
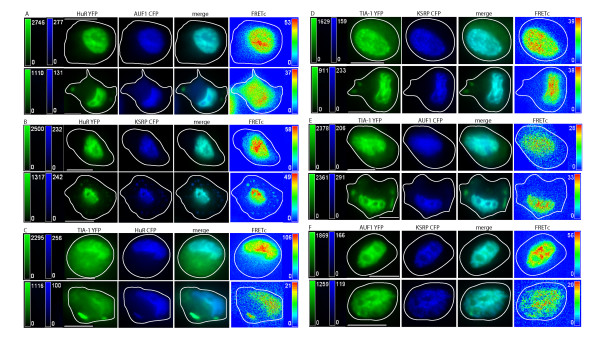
**Localization and FRET interactions of exogenous ARE binding proteins upon oxidative stress**. Vector constructs of ARE binding proteins labeled with eYFP or eCFP were transiently transfected into DDT1-MF2 cells. Cells were treated with 0.5 mM sodium arsenite for 30 minutes. The localization of ARE-BPs is shown in control cells (top row), and sodium arsenite treated cells (bottom row), in each panel of images. Images in each row, from left to right, show the localization of an ARE-BP detected via: i) YFP in green, ii) CFP in blue, iii) merged YFP and CFP images, iv) FRETc signal in a thermal pseudocolor scale. A color matched signal intensity scale is indicated for each image. All scale bars are 10 μm in length. Cell outline as shown. **Panel A: **HuR-YFP and p37AUF1-CFP. **Panel B: **HuR-YFP and KSRP-CFP. The signal intensity of the KSRP-CFP image (bottom row) was increased to more easily view SGs. **Panel C: **TIA-1-YFP and HuR-CFP. **Panel D: **TIA-1-YFP and KSRP-CFP. Images are of live cells under DMEM. **Panel E: **TIA-1-YFP and p37AUF1-CFP. A very small portion of p37AUF1 is detectable in SGs, (elongated SG, lower right side of cell). **Panel F: **p37AUF1-YFP and KSRP-CFP.

In a previous report [15], we investigated the localization and interaction of fluorescently labeled HuR and p37AUF1 proteins. Herein, we expand this examination to include KSRP and TIA-1, both exogenous and endogenous, thereby increasing substantially the number of permutations of interacting proteins to be examined. As was shown previously, and now in Figures 1 and 2, under basal conditions, HuR and AUF1 are predominantly nuclear, a pattern consistent for both endogenous and exogenously produced proteins. Under basal conditions, KSRP expression is also predominantly nuclear, a result consistent for both endogenous and exogenous tagged proteins [13,36], Figures 1 and 2. As documented previously [37-39], and now herein, in unstressed cells, TIA-1 is expressed uniformly throughout the nucleus and cytoplasm, Figures 1 and 2. Interestingly, all four ARE-BPs appear to be excluded from the nucleoli. Localization and interaction of all RNA-BPs in response to various stimuli is summarized in Table 1.

**Table 1:** Cellular Location of ARE Binding Proteins

Endogenous Proteins					
Protein	Nucleus	Cytoplasm	SG (Arsenite)	PB	Anisomycin

AUF1	+++	+	+	-	N to C

HuR	+++	++	+++	-	N to C

KSRP	+++	+	+	-	N to C

TIA-1	+++	+++	+++	+	NA^a^

**Exogenous proteins**					

AUF1	+++	-	+	-	N to C

HuR	+++	+	+++	-	N to C

KSRP	+++	-	+	-	N to C

TIA-1	+++	+++	+++	NT^b^	NC

a) NC, no change, b) NT, not tested

### Localization of endogenous ARE binding proteins to cytoplasmic Stress Granules

Cytoplasmic SGs have been described as sites of triage for RNA transcripts when cells are undergoing stress [40-43]. It is generally accepted that SGs contain proteins associated with mRNA translation initiation, translation control, and to some degree, with mRNA decay [44]. Upon initiation of cellular stress, these proteins, as well as a vaguely described subset of mRNA molecules, rapidly relocalize to SGs. SGs have also been shown to be dynamic structures, originating as smaller punctate bodies that can ultimately fuse to form larger, irregularly shaped structures [45]. They have also been shown to be in dynamic contact, exchanging components with PBs [43].

As demonstrated by the relocalization of TIA-1, induction of oxidative stress with arsenite induces the rapid formation of cytoplasmic SGs [15]. Similarly, herein, TIA-1 has been shown to rapidly relocate to SGs in DDT1-MF2 cells within 30 minutes post arsenite treatment, Figures 1 and 2, Panels C, D and E, Row 2 (of each panel). HuR also rapidly relocalizes to SGs [46], Figures 1 and 2, Panels A, B and C, Row 2 (of each panel). Unlike TIA-1, HuR is likely relocalizing from the nucleus to the cytoplasm and then to SGs whereas TIA-1 is already in the cytoplasm prior to the initiation of oxidative stress. Further, we demonstrate that KSRP and AUF1 relocalize from the nucleus to cytoplasmic SGs with rapid time frame, similar to that of HuR [14], Figure 1, Panels B, D and F, Row 2 for KSRP and Figure 1, Panels A, E and F, Row 2 for AUF1. In images of arsenite treated cells, it should be readily apparent that although TIA-1, HuR, AUF1 and KSRP all populate SGs, the proportion of each protein that relocalizes to SGs varies considerably with the relocalization of TIA-1 being the most robust. Based on relative fluorescent intensity, the concentration of endogenous TIA-1 present in SGs, post arsenite, is noticeably greater, in general, than in either the nucleus or cytoplasm. Although still marked, HuR shuttles to a lesser degree than TIA-1 with more HuR protein remaining in the nucleus than is present in the SGs. For KSRP and AUF1, only a small portion of total cellular protein relocalizes to SGs.

Figure 1 also documents the interaction of ARE-BPs present within SGs. Panel A, Row 2 shows the colocalization and interaction of HuR and AUF1 in DDT1-MF2 cells after treatment with arsenite. The merged view shows that HuR is likely to be at a higher concentration in SGs than is AUF1, whereas the two proteins are more or less equally abundant in the nucleus, as demonstrated previously by western blotting [15]. The FRETc image shows that HuR and AUF1 are in stable contact in the nucleus and in SGs. As the peak intensity of the FRETc signal is proportional to the proximity and amount of fluorophors present, a lower intensity FRETc signal from the presumably lesser quantity of AUF1 present in SGs is to be expected (i.e., the concentration of AUF1 is limiting).

Figure 1, Panel B, Row 2 shows the colocalization and interaction of KSRP and HuR. As stated previously, both HuR and KSRP are present dominantly in the nucleus, to a small extent in the cytoplasm, and under conditions of oxidative stress, in SGs. In the merged view, it is easy to see that KSRP and HuR colocalize in the nucleus and to a smaller extent the cytoplasm. Colocalization within SGs is much less readily detectable. This, again, is presumably due to the much lower concentration of KSRP in SGs compared to that of HuR. The FRETc view demonstrates that there is close and stable interaction between HuR and KSRP in the nucleus and to a small extent in cytoplasm and SGs. That being said, the SG FRETc signal is quite distinct if image intensity is adjusted to permit visualization.

Figure 1, Panel C, Row 2 shows the colocalization and interaction of TIA-1 and HuR in arsenite treated cells. Both TIA-1 and HuR are present in the nucleus and in SGs. Under these conditions, TIA-1 is present in SGs and cytoplasm to a much greater extent than is HuR. In the merged view, it is readily apparent that HuR is dominant in the nucleus and TIA-1 dominant in SGs. A FRETc signal is present in the nucleus but is much stronger in the SGs and is indicative of close and stable contact of proteins in high relative abundance.

Figure 1, Panel D, Row 2 shows the localization and interaction of KSRP and TIA-1. Under stress conditions, both KSRP and TIA-1 relocalize to SGs. The merged image shows that, relatively speaking, more KSRP is in the nucleus and more TIA-1 is in SGs. The FRETc image shows a close and stable contact between KSRP and TIA-1 in SGs and nucleus.

Figure 1, Panel E, Row 2 shows the colocalization and interaction of AUF1 and TIA-1. Both TIA-1 and AUF1 are present in nucleus and SGs. The merged view shows that the TIA-1 signal intensity is far greater in SGs than is AUF1 but that they are both readily detectable within the nucleus. A FRETc signal is present in the nucleus and in SGs and shows the close and stable interaction of TIA-1 and AUF1 in both of these locations.

Figure 1, Panel F, Row 2 shows the localization and interaction of KSRP and AUF1. Both KSRP and AUF1 are present in the nucleus and only a small portion of either of these proteins moves to SGs. The merged view shows the colocalization of KSRP and AUF1 to nucleus and SGs. A FRETc signal is present in both the nucleus and SGs indicating that these proteins are in a close and stable interaction.

In previous experiments, as well as in the current Figure 2 representing exogenous proteins, we were unable to detect AUF1 in SGs [15]. Supporting our previous conclusion was an absence of literature describing the localization of AUF1 to SGs. On average, the AUF1 signal intensity present in SGs is about 1/10th of the maximum fluorescence intensity present in the cell, with the most intense signal always being within the nucleus. Increasing the overall signal intensity allowed the detection of a weak fluorescence signal for AUF1 when present in SGs (Figure 1). It should be noted that our previously reported results were based solely on expression of the p37 splice form of AUF1. In contrast, under the currently used experimental conditions, immunostaining of endogenous AUF1 protein detects all 4 splice-forms (37, 40, 42 and 45), the results therefore being representative of the totality of intracellular AUF1 protein.

Pertaining to KSRP, the reason(s) underlying the variability of exogenous protein expression in SG is currently unknown. Two potential explanations might be level of KSRP protein expression or epitope tagging of the protein. The non-uniformity of response between exogenous and endogenous protein demonstrates the utility of immuno-FRET over transient transfection in studying cellular protein localization.

### Endogenous ARE binding protein localization to Processing Bodies

**Figure 3:**
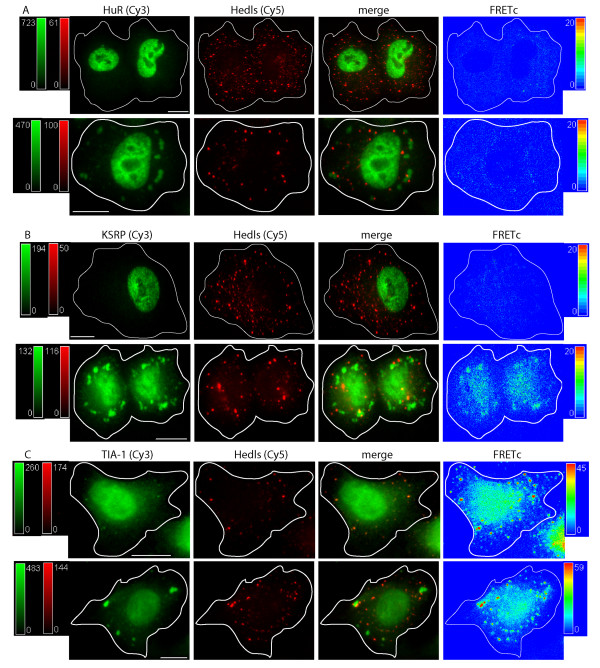
**Localization and FRET interactions of endogenous ARE binding proteins upon oxidative stress: P bodies and Stress Granules**. ARE-BPs were detected via immunocytochemistry. Cells were treated with 0.5 mM sodium arsenite (SA) for 30 minutes. PBs were defined by detection of Hedls. The localization of each ARE-BP and Hedls is shown in control cells (top row), and SA treated cells (bottom row), in each panel of images. Images in each row, from left to right, show: i) the ARE binding protein detected via a Cy3 labeled 2° Ab in green, ii) Hedls detected via a Cy5 labeled 2° Ab in red, iii) merged Cy3 and Cy5 image, iv) FRETc signal in thermal pseudocolor scale. The images were captured at the level of the nucleus. A color matched signal intensity scale is indicated for each image. All scale bars are 10 μm in length. **Panel A: **HuR and Hedls. The Cy5 intensity in the Hedls and merged images (top row) was increased for easier detection of the cytoplasmic PB. The FRETc signal was set to an intensity of 20 to indicate the lack of a specific protein interaction as an intensity of < 20 is considered nonspecific. **Panel B: **KSRP and Hedls. The Cy5 intensity in the Hedls and merged images (top row) was increased for easier detection. The FRETc signal was set to an intensity of 20 to indicate the lack of a specific protein interaction. **Panel C: **TIA-1 and Hedls.

PBs are generally recognized as sites associated with RNA degradation. They are distributed throughout the cytoplasm and contain numerous proteins involved in RNA decay [32,47,48]. Interestingly, PBs appear to be constitutively present in mammalian cells but need to be induced in yeast [43]. In the current studies, we were interested in determining whether or not ARE-BPs colocalize to PBs and if so, could interactions of proteins generally assumed to be in these intracellular compartments, be detected. Figure 3, Panel A, Row 1 details the intracellular localization of HuR and Hedls. As before, in unstimulated cells, HuR is localized predominantly to the nucleus whereas Hedls is localized diffusely in the cytoplasm as well as in obvious PB structures. The merged view shows that there is no colocalization of HuR and Hedls, a finding concordant with a lack of detectable FRET. A similar result is found for the (lack of) interaction between KSRP and Hedls, Figure 3, Panel B, Row 1.

Figure 3, Panel C, Row 1 details the results for interactions between TIA-1 and Hedls. TIA-1 demonstrates a nuclear and cytoplasmic distribution that includes punctuate bodies in the cytoplasm whereas Hedls is present in both PBs and generally in the cytoplasm. Interestingly and importantly, the FRETc view demonstrates a unique interaction between TIA-1 and Hedls delimited to PBs. This finding is concordant with previous descriptions delineating the dynamic nature of PBs and their potential for interaction (protein exchange) with SGs [49].

To investigate the potential for interaction of proteins compartmentalized to SGs and PBs, SGs were first induced by oxidative stress. Figure 3 indicates that the morphology of PBs, as identified by the cellular localization of Hedls, is unaffected by oxidative stress or by the formation of cytoplasmic SGs. This result has been recapitulated using Dcp1a as another marker of PBs [50], data not shown. Further, oxidative stress induced relocalization of ARE-BPs to SGs but did not alter the interaction of each specific protein with Hedls when present in PBs. Interaction between SGs and PB proteins can be seen in the merged view of Figure 3, Panel C, Row 2. To examine this in greater detail, the interaction of SGs and PBs is seen via 3D reconstruction collected along the Z-axis, (Additional file 1). The 3D reconstruction of KSRP/Hedls interaction demonstrates that SGs and PBs are not cytoplasmic spheres but rather, elongated oval structures with substantial depth throughout the cytoplasm. Of note is the observation that more than one PB can be in contact with a single SG. This image also shows that an observed protein/protein colocalization does not necessarily lead to a FRET detectable interaction. Figure 3 Panel C Row 2 demonstrates a FRET signal is generated for the interaction of TIA-1 and Hedls. This result strongly supports the conclusion that the TIA-1/Hedls interaction is specific and not secondary to a packing artifact.

TIA-1 was detected in PBs during both unstimulated and MAPK stimulated conditions. In unstimulated cells, a portion of the cytoplasmic TIA-1 appears to be present in PBs leading to the generation of a FRET signal between TIA-1 and Hedls. In stressed cells, TIA-1 is present in both SGs and PBs. In this case, the strongest FRET signal was generated at the interface of TIA-1 containing SGs and Hedls containing PBs. Why TIA-1, along with TTP [43,51] and BRF1, [43] are present in PBs, but HuR, AUF1 and KSRP are not, is currently unknown. The potential for interaction between AUF1 and Hedls (or Dcp1a) was unable to be investigated, as the only antibodies available to detect these proteins were rabbit polyclonal.

### Cellular localization of ARE RNA binding proteins: effect of MAP Kinase activation

**Figure 4:**
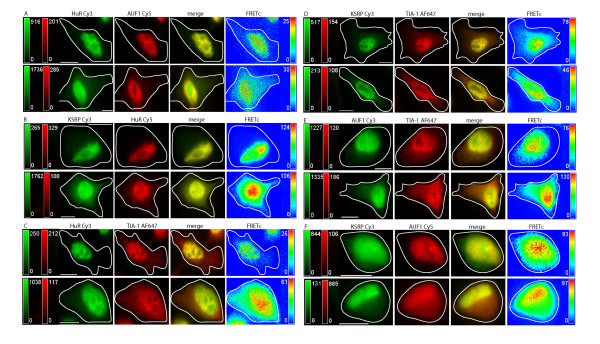
**Localization and FRET interactions of endogenous ARE binding proteins upon MAPK activation**. ARE-BPs were detected via immunocytochemistry. Cells were treated with 75 nM anisomycin for 24 hours. The localization of the ARE-BPs is shown in control cells (top row), and anisomycin treated cells (bottom row), in each panel of images. Images in each row, from left to right, show the localization of an ARE-BP detected via: i) a Cy3 labeled 2° Ab in green, ii) a Cy5 or AF647 labeled 2° Ab in red, iii) merged Cy3-Cy5 image, iv) FRETc signal in a thermal pseudocolor scale. A color matched signal intensity scale is indicated for each image. All scale bars are 10 μm in length. Cell outline as shown. **Panel A: **HuR (Cy3) and AUF1 (Cy5). **Panel B: **KSRP (Cy3) and HuR (Cy5). **Panel C: **HuR (Cy3) and TIA-1 (AF647). **Panel D: **KSRP (Cy3) and TIA-1 (AF647). **Panel E: **AUF1 (Cy3) and TIA-1 (AF647). **Panel F: **KSRP (Cy3) and AUF1 (Cy5).

**Figure 5:**
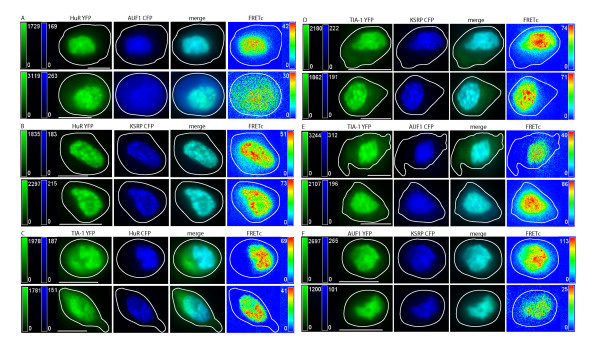
**Localization and FRET interactions of exogenous ARE binding proteins upon MAPK activation**. ARE binding proteins were detected via transient transfection of labeled vector constructs into DDT1-MF2 cells. Cells were treated with 75 nM anisomycin for 24 hours. The localization of ARE-BPs is shown in control cells (top row), and anisomycin treated cells (bottom row), in each panel of images. Images in each row, from left to right, show the localization of an ARE-BP detected via: i) YFP in green, ii) CFP in blue, iii) merged YFP and CFP images, iv) FRETc signal in a thermal pseudocolor scale. A color matched signal intensity scale is indicated for each image. All scale bars are 10 μm in length. Cell outline as shown. **Panel A: **HuR-YFP and p37AUF1-CFP. **Panel B: **HuR-YFP and KSRP-CFP. **Panel C: **TIA-1-YFP and HuR-CFP. **Panel D: **TIA-1-YFP and KSRP-CFP. **Panel E: **TIA-1-YFP and p37AUF1-CFP. **Panel F: **p37AUF1-YFP and KSRP-CFP.

Stimulation of MAP kinase pathways has the biochemical effect of stabilizing ARE containing RNAs [52-54]. MAPK stimulation also has the effect of intracellular redistribution of several ARE binding proteins. Upon activation, both HuR and AUF1 are relocalized from the nucleus to a combined nuclear/diffuse cytoplasmic distribution, Figures 4 and 5. However, as shown previously [15], the time course of redistribution for the two proteins is markedly different. When stimulated with anisomycin, HuR shuttles from nucleus to cytoplasm in < 90 minutes whereas movement of AUF1 requires on the order of 24 hours (both visually and by Western Blot). This disparate time frame may underlie the biological plausibility of the opposing nature of these ARE-BPs, that is, the rapid stabilizing influence of HuR on ARE containing RNAs.

In the current study, we extended this exploration to examine the effects of MAPK stimulation on KSPR and TIA-1. As TIA-1 possesses a general cytoplasmic distribution in unstimulated cells, we speculated that MAPK stimulation might not have a discernable effect on its distribution and indeed, it does not, Figures 4 and 5, Panels C, D and E. Figure 4 summarizes the results of pair-wise endogenous protein/protein interactions following MAPK activation by anisomycin treatment of DDT1-MF2 cells. In each case, the upper panel is representative of control, vehicle treated cells, whereas the bottom panel is representative of anisomycin treated cells (24 hr exposure to 75 nM). As can be seen, in Figure 4 Panels B, D, and F, MAPK activation induces nucleocytoplasmic shuttling of KSRP. In both the merged and FRET image panels, relocalization of endogenously expressed protein pairs to the cytoplasm demonstrates FRET between KSRP/TIA-1, KSRP/HuR, and KSRP/AUF1. Comparing Figure 4 to Figure 5, variability exists in the response of endogenous versus exogenous KSRP to MAPK activation. No shuttling of exogenous KSRP is seen due to MAPK stimulation, compared to readily detectable shuttling of the endogenous proteins.

Using FRETc, we are able to demonstrate that all of the ARE-BPs studied interact in all the pair-wise combinations tested when localized to the nucleus and in the cytoplasm when relocalization is due to MAPK stimulation. These results are supportive of the notion that ARE-BPs are bound to multiple AREs as part of multiprotein complexes. One implication of this conclusion is that fine-tuning of the half-life of an RNA molecule is almost certainly tightly regulated by a large number of factors and conditions that remain to be elucidated.

### MAP Kinase stimulation: movement to Processing Bodies or not?

**Figure 6:**
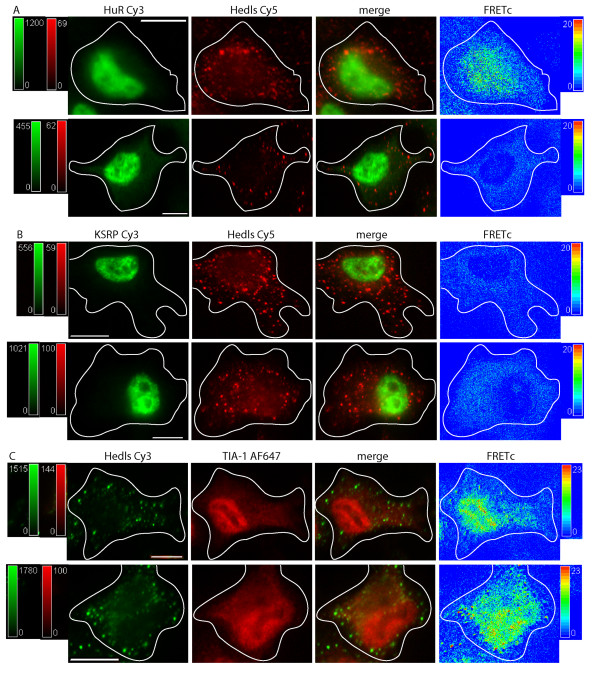
**Localization and FRET interactions of endogenous ARE binding proteins upon MAPK activation: Movement to P bodies? **ARE-BPs were detected via immunocytochemistry. Cells were treated with 75 nM anisomycin for 24 hours. PBs were defined by presence of Hedls. The localization of each ARE-BP and Hedls is shown in control cells (top row), and anisomycin treated cells (bottom row), in each panel of images. Images in each row, from left to right, show the localization of a protein detected via: i) a Cy3 labeled 2° Ab in green, ii) a Cy5 or AF647 labeled 2° Ab in red, iii) merged Cy3-Cy5 image, iv) FRETc signal in a thermal pseudocolor scale. A color matched signal intensity scale is indicated for each image. All scale bars are 10 μm in length. Cell outline as shown. **Panel A: **HuR (Cy3) and Hedls (Cy5). The FRETc signal was set to an intensity of 20 to indicate the lack of a specific protein interaction. A FRETc signal intensity less than 20 was considered nonspecific. The Cy5 signal intensity was increased in all images of Hedls for better visualization. **Panel B: **KSRP (Cy3) and Hedls (Cy5). The FRETc signal was set to an intensity of 20 to indicate the lack of a specific protein interaction. The Hedls Cy5 signal intensity was increased in all images for ease of PB detection. **Panel C: **Hedls (Cy3) and TIA-1 (AF647).

Figure 6 examines the effect of MAPK stimulation on PBs and the localization of the ARE-BPs currently being investigated. As MAPK stimulation and inhibition has been shown to reciprocally stabilize and destabilize a number of ARE containing RNAs [55], and degradation of mRNA has been associated with protein components of SGs and PBs [32,43,48,56], we hypothesized that certain ARE-BPs may differentially localize to PBs. As was shown in Figures 4 and 5, upon MAPK stimulation, HuR, AUF1 and KSRP all move from the nucleus to the cytoplasm with the extant cytoplasmic localization of TIA-1 being unaffected. Figure 6, Panels A, B and C, demonstrate that the morphology and location of PBs, as defined by Hedls, appears entirely unaffected by MAPK stimulation. We have also shown this to be true for Dcp1a (data not shown).

In Figure 6, Panel A, HuR and Hedls did not colocalize nor was FRET in evidence. Similarly, Figure 6, Panel B demonstrates that MAPK activation did not cause KSRP and Hedls to interact. However, Figure 6, Panel C shows that the Hedls/TIA-1 do interact, a result consistent with that shown in Figure 3, Panel C. As the cellular localization of both the TIA-1 and Hedls are apparently unaffected by MAPK stimulation, it follows that their interaction may also unaltered by this stimulation. Thus, TIA-1 and Hedls show a FRETc detected interaction in both unstimulated and stimulated conditions.

### Processing Body components localize to *in vitro *transcribed RNA

**Figure 7:**
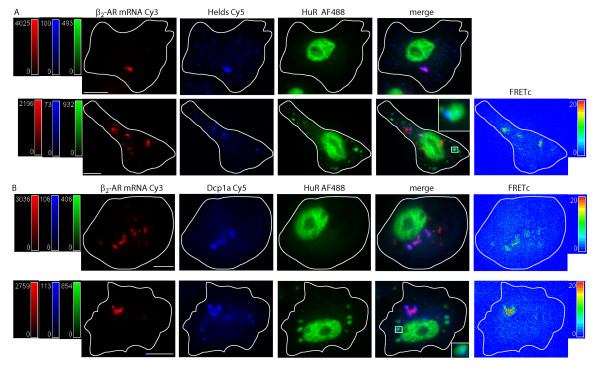
**Interaction of Processing body components Hedls and Dcp1a with ARE containing mRNA**. 2'F stabilized, Cy3-labeled β-AR mRNA was transiently transfected into DDT1-MF2 cells. The cells were subjected to oxidative stress (30 minute exposure to 0.5 mM sodium arsenite (SA)) and the proteins were detected by immunocytochemistry. Top row, control cells, bottom row, SA treated cells. Images in each row, from left to right, show the localization of: i) the Cy3-labeled mRNA in red, ii) a Cy5-labeled 2° Ab in blue, iii) HuR detected via an AF488 labeled 2° Ab in green, iv) merged Cy3, Cy5 and AF488 image, v) Cy3-Cy5 FRETc signal in a thermal pseudocolor scale. A color matched signal intensity scale is indicated for each image. All scale bars are 10 μm in length. Cell outline as shown. **Panel A: **β-AR mRNA (Cy3), Hedls (Cy5), and HuR (AF488). The inset in the merged view shows a close up of the SGs (green) and PBs (blue) interaction. No FRETc image presented due to the disparity of the Cy3/Cy5 signal intensity. **Panel B: **β-AR mRNA (Cy3), Dcp1a (Cy5), and HuR (AF488). The inset in the merged view shows the interaction between SGs (green) and PBs (blue).

Direct investigation of ARE-containing RNA molecules allows us to begin to interconnect data for ARE-BP localization with specific RNAs. Currently, overcoming the obstacle of visualizing RNA molecules with short half-lives can be approached in one of two ways: plasmid based expression of RNAs encoding stem loops recognized by the MS2-coat protein [57] or *in vitro *transcription and transfection of fluorescently labeled RNAs with or without 2'F (dUTP) chemical stabilization. For this set of experiments, we chose the latter approach. To visualize target RNA, full length, 2'F-dUTP modified, 5' capped, polyadenylated, hamster β_2 _adrenergic receptor (β-AR) RNA was *in vitro *transcribed, utilizing an appropriate stoichiometry of Cy3-labeled UTP. Importantly, by nondenaturing gel shift assay, the fully modified RNA was able to bind ARE-BPs with normal (low nM) affinity (data not shown). As is evident in Figure 7, the transfected Cy3-labeled, ribonuclease resistant RNA accumulated in punctate cytoplasmic structures. We hypothesized that since 2'F-modified RNA is generally not translationally competent, it may undergo sequestration into translationally silent compartments, i.e., SGs or PBs. Nonetheless, we wished to determine whether or not the ARE was accessible and would bind ARE-BPs under resting and stimulated conditions. Figure 7, Panel A, Row 1 shows a DDT1-MF2 cell containing the Cy3-labeled RNA, which was immunostained for Hedls and HuR. The merged view demonstrates that Hedls colocalizes with the Cy3-labeled RNA as well as being present in small, punctate PB-like structures. In this instance, no FRET data was able to be collected due to a disparity in fluorescent signal intensities.

Figure 7, Panel A, Row 2 shows the effect of oxidative stress on the Cy3-labeled RNA in conjunction with Hedls and HuR. Image 1 shows the mRNA by itself. Image 2 confirms that oxidative stress does not affect Hedls localization. Image 3 shows that HuR readily relocalizes to SGs in the presence of the introduced mRNA. Image 4 shows the merged view with colocalization of Hedls and the Cy3-labeled mRNA. Image 5 shows the FRET detectable interaction between Hedls and the mRNA.

We speculate that what underlies this result is the ribonuclease resistant RNA is attracting or sequestering Hedls protein. Figure 7, Panel B shows the same experiment but this time with the PB component, Dcp1a, which was detected by immunostaining (in addition to HuR). Similar results were obtained as outlined in Figure 7, Panel A; Dcp1a is present in the cytoplasm in PBs but also colocalizes with Cy3-labeled RNA. Image 4 of Row 2, Panel B shows the merged view. HuR is present in SGs but Dcp1a and the RNA are not. The RNA and Dcp1a are in close and stable contact as indicated by the FRETc image. This result, consistent with that found with Hedls, shows that Dcp1a recognized and interacted with the stabilized mRNA molecule in a location associated with RNA degradation.

## Discussion

It has long been recognized that certain RNA-BPs undergo nuclear/cytoplasmic shuttling and are capable of localizing in discrete intracellular compartments. Based on the seminal work of Anderson and others [42,43,56,58], it is also well documented that certain RNA-BPs can colocalize. However, colocalization, as identified by microscopy and/or by co-immunoprecipitation, can be limited in interpretation. More precisely, colocalization identified by conventional and/or confocal microscopy can be vague and does not necessarily permit the conclusion that two proteins that appear to colocalize are actually in sufficiently close proximity to physically or functionally interact. This is also true for immunoprecipitation experiments that generally do not necessarily distinguish between direct and indirect protein/protein interaction. Therefore, one objective of the current work was to identify the intracellular localization and potential interactions of several key RNA binding proteins, both endogenous and exogenous, using FRET. Although a positive FRET signal does not guarantee that proteins are interacting functionally, it does provide strong evidence that two (or more) proteins are in sufficiently close proximity to physically interact.

Although immuno-FRET of ARE binding proteins is being described herein for the first time, there is certainly ample precedent demonstrating the usefulness and veracity of this methodology [31,59-62]. A major consideration in analyzing immuno-FRET data generated with fluorescently labeled antibodies (primary or secondary), is an extraordinary attention to detail necessary to obviate issues related to background signal and non-specific fluorescence. For example, certain antibodies (and cell types) demonstrate high levels of background or non-specific fluorescence that can constitute a major limitation. Another obvious limitation of immuno-FRET is the increased probability of a false-negative signal due to the position and/or orientation of one secondary antibody fluorophor relative to the other. As with any FRET measurement, the acquired raw fluorescence data must be corrected by subtracting the appropriate channel bleed-through determined specifically for the experimental system being used.

In previous investigations [15], we demonstrated that p37AUF1/hnRNP D and HuR, were colocalized in both nucleus and cytoplasm and physically interacted, as detected by FRET. Additionally, and in support of existing biochemical data [63-65], we demonstrated that p37AUF1 and HuR can both homo and heterodimerize [15]. The current study extends the identification of heterologous permutated interactions to include those between AUF1, HuR, KSRP, TIA-1, and Hedls, proteins variably associated with increased RNA turnover, RNA stabilization, and translational suppression. Our current findings demonstrate that both endogenous and exogenous, fluorescently-tagged AUF1, HuR, TIA-1 and KSRP are dominantly nuclear, Figures 1 and 2, and that each protein pair, AUF1/HuR, KSRP/AUF1, TIA-1/HuR, KSRP/TIA-1, AUF1/TIA-1, and KSRP/HuR, demonstrates nuclear FRET. This is in contrast to the lack of FRET between each of these ARE-BPs and β-tubulin that was used as a negative control for immuno-FRET (data not shown).

Induction of oxidative stress with arsenite causes proteins to aggregate in SGs to varying degrees with further distinctions between endogenous and exogenous proteins. Specifically, arsenite-induced stress caused rapid formation of cytoplasmic SGs with the presence of endogenous HuR, AUF1, KSRP and TIA-1 being readily detected. However, robust FRET signals in SGs were limited to the interactions between TIA-1/HuR and TIA-1/KSRP and to a lesser extent, TIA-1/AUF1, HuR/AUF1, KSRP/AUF1 and KSRP/HuR. Interestingly, and in contrast to their endogenous counterparts, the exogenous pair of TIA-1-YFP/AUF1-CFP does not demonstrate a readily detectable FRET signal.

The presence and interaction all four ARE-BPs in SGs may seem anomalous given that they have putatively different roles when bound to an ARE. When preceding experiments failed to detect AUF1 in SGs, we initially reasoned that this result was consistent with the biology of AUF1 generally being considered an mRNA destabilizing protein; hence, it might not be expected to be bound to an RNA being sequestered and stabilized within an SG. This reasoning could be extended to KSRP. However, in biochemical studies, we have shown that AUF1 and HuR are able to bind to each other and to co-reside on the β_2_-adrenergic receptor ARE [15]. Thus, the notion that a protein, based on putative function, would or would not localized to a compartment with a putative antithetical function does not appear to be valid. For this to be true, rapid movement of an mRNA molecule to SGs, upon the initiation of cellular stressor, would require a screening mechanism to be present upon 'entrance' to the SG such that it would remove all 'destabilizing' protein entities. This does not appear to be occurring as the formation of SG and hence removal of all non-essential mRNA from active translation in the cytoplasm during a stress response appears to take precedence.

Potentially adding to the uncertainty of ARE-BP localization and interactions is the discrepancy, upon cellular activation, between endogenous and exogenous proteins. As our studies show, this appears to be the case for KSRP. This particular result underscores one of the potential problems associated with transient transfection of exogenous proteins and the advantage, when possible, of visualizing endogenous proteins by immuno-FRET.

An unanticipated finding was the strong FRET signal between endogenous TIA-1 and Hedls, Figure 3. The FRET signal between TIA-1 and Hedls seen when the proteins are present in juxtaposed SGs and PBs respectively, is supported by the work of Anderson, Kedersha and colleagues, demonstrating that certain constituent RNA binding proteins and other compositional constituents of SGs and PBs can undergo dynamic interchange [38,43,45,56]. Potentially underlying the interchange of SG and PB components is translationally silenced RNA and its associated RNP complexes, some of which appear to be present in both PBs and SGs.

Studies indicate that mRNAs cycle between PBs and SGs, depending on the availability of translation initiation or degradation machinery. A working model, called the mRNA cycle, was proposed by Balagopal and Parker [66]. In this model, transcribed mRNAs initially undergo subsequent rounds of translational initiation, elongation and termination, producing polypeptides until a change in the cellular environment alters this cycle directing the mRNAs away from ribosomes and towards SGs and PBs. What is undoubtedly a complex and as yet unknown pathway is that determining whether a stalled mRNA will differentially populate PBs or SGs.

As RNAs are dynamic between PBs and SGs so then too are the proteins associated with them. The complete proteomic composition of PBs and SGs is still being elucidated, however, a number of proteins have been identified and localized to one or both of these cytoplasmic granules [67]. Relevant to our work, TIA-1 (and TIAR) nucleates SGs but is not found in PBs; in contrast, Hedls and Dcp1 are found in PBs but not in SGs. More RNA binding proteins have been found to be common to both SGs and PBs than exclusively present in one or the other. This might indicate that the proteins common to SGs and PBs play a more general role in the maintenance/structure of an mRNA molecule while present in a particular granule and those RNA BPs specific to SGs or PBs have a role in the fate of the mRNA in the granule. Movement of mRNPs from polysomes to SGs or PBs may be attributed to different mRNP conformational states due to rearrangement, presence or exchange of ARE-BPs on specific RNAs so our finding that all ARE-BP studied are present in SGs may, in reality, have not been so unexpected.

A somewhat disappointing finding was the more or less general, diffuse effect of MAP kinase stimulation on the relocalization of RNA binding proteins, Figures 4 and 5. As demonstrated previously, anisomycin causes a rapid shuttling of HuR from nucleus to cytoplasm, a result consistent with the observation that MAPK activation is widely recognized to cause stabilization of ARE containing mRNAs [55]. In contrast, AUF1 and KSRP shuttle from nucleus to cytoplasm with a considerably longer kinetic. Unlike treatment with arsenite, MAPK activation does not cause SG formation. Instead, the cytosolic presence of HuR, AUF1, and KSRP are detectably increased but remain diffuse with a peri-nuclear to cell periphery decreasing expression gradient. As in the nucleus, each of these pairs of proteins exhibits FRET with the relative intensity decreasing along their concentration gradients. Consistent with arsenite studies, MAPK activation does not produce a visibly detectable interaction between Hedls and either KSRP or HuR.

There is also biochemical evidence in the literature that MAPK activation causes the relocalization of RNA and associated RNA binding proteins in a phosphorylation-dependent manner [13,68-73]. For example, in the case of HuR, several kinases appear to regulate its intracellular localization. Kim, et. al. [74], have described nuclear retention of HuR promoted by Cdk1 mediated phosphorylation (S202). HuR is also phosphorylated at a number of other residues: S88, S100, and T118 by checkpoint kinase 2 (Chk2) [23,75], at S158 by PKCα, at S221 and S318 by PKCδ [25,69-71] and at S242 by an as yet unidentified kinase [74]. The location of the modified residues are either in or between two of the three RNA recognition motifs, (RRM 1 and 2), or within the nucleocytoplasmic shuttling sequence (205-237) of HuR [4]. The effect of phosphorylation at S202 and S242 is nuclear retention whereas phosphorylation of S158, S221, and S318 leads to nuclear export of HuR. Phosphorylation at S88, S100 and T118 does not appear to affect the localization of HuR. In addition, phosphorylation at S88, T118 [23,75], S158, S221 [70] or S318 [25] was shown to enhance the binding of HuR to target AREs with S100 phosphorylation showing the opposite affect [23,75] and S221 phosphorylation no effect [25] on HuR binding. HuR is also modified by methylation at R217 by CARM1, a change that was implicated in cytoplasmic shuttling of HuR in response to lipopolysaccharide [76]. The phosphorylation of S202 lead to nuclear retention of HuR by allowing binding of 14-3-3θ to this phospho-form of this protein [74].

In addition to HuR, phosphorylated KSRP [26], TTP [77], AUF1 [78] and BRF1 [79], have all been reported to bind a member of the 14-3-3 protein family in either the nucleus or cytoplasm. This may be a general method of alteration of ARE binding protein function and localization and is consistent with the cellular functionality of 14-3-3 proteins, in general [80].

In a related experimental direction, we attempted to address a long-standing interest, that of colocalization of ARE-containing mRNAs and RNA binding proteins. To perform these experiments, *in vitro *transcribed, capped, polyadenylated, chemically stabilized (2'F) fluorescently labeled (Cy3-UTP) RNA was transfected into DDT1-MF2 cells. Two outcomes are noted. First, that the 2'F/Cy3 RNA is remarkably stable demonstrating virtually no change in abundance at 24 hrs post transfection. Secondly, the transfection of RNA forms distinct cytoplasmic aggregates. This finding is entirely consistent with that of Barreau et al [81], where aggregates of a similar description were noted. However, these authors conclude that the aggregates may be secondary to a stress response simply due to the presence of excess RNA. Our finding is also similar to that of Wilkie and Davis [82], who described, in *Drosophila*, the localization and trafficking of capped, fluorescently labeled *run *RNA.

Going beyond this observation, we used immuno-detection of the decapping proteins, Dcp1 and Hedls, to demonstrate that the aggregated RNA and the decapping enzymes were colocalized. In fact, we were able to observe robust FRET between Cy3-labeled β-AR mRNA and Dcp1. These results lead to a couple of conclusions. First, that interaction of labeled RNA and protein can be observed, a potentially highly useful proof of principal. Secondly, that transfection of *in vitro *transcribed, chemically stabilized RNA is probably trapped in PB due to its inability to undergo appropriate degradation. In contrast, FRET was not observed between labeled RNA and the ARE binding protein, HuR. This is perhaps not surprising given the absence of HuR in PBs.

At least two other papers have been published describing FRET between RNA and RNA binding proteins. Lorenz [83] infers FRET by a reduction in fluorescence lifetime (FRET-FLIM) secondary to the interactions of both Venus-PTB and YFP-Raver1 proteins and generic SytoxOrange labeling of RNA. Huranova, et. al. [84], describe FRET-FLIM between eCFP-tagged hnRNP H bound to an engineered high-affinity consensus binding site within the RNA and eYFP-tagged MS2 coat protein bound to its cognate stem-loop binding site. The advance to the field from the data presented here in, is the demonstration that a specific labeled RNA molecule can interact with a specific RNA binding protein and that their interaction can be detected within a specific intracellular compartment. Thus, alternative approaches, each with their relative strengths and weaknesses are increasingly becoming available.

## Conclusions

In summary, we have demonstrated herein that the interaction between a number of RNA binding proteins, both endogenous and exogenous, can be detected by FRET. Further, there is a reasonable degree of concordance between the shuttling and intracellular localization of endogenous and exogenous ARE-BPs, with exceptions duly noted. There is also substantial concordance of the intracellular localization of ARE-BPs by imaging and biochemical techniques. Future tests of usefulness and validity of these methods will invariably involve knockdown experiments and site directed mutagenesis of ARE-BPs to examine factors such as phosphorylation state as a modulator of localization and protein/protein interaction. Our current work cannot determine whether the interactions we detect are the primary or the most functionally relevant interactions of any given RNA-BP. Additional methods, such as 3-FRET [85] may allow us to begin to address these important questions.

## Methods

### Vector Constructs

Construction of the HuR-eYFP, HuR-eCFP, p37AUF1-eCFP and p37AUF1-eYFP vectors was as described previously [15]. An Xho1/Kpn1 fragment of KSRP was subcloned into pECFP.C1 (Clonetech) yielding the KSRP-eCFP vector construct. Similarly a Bgl II/EcoRI fragment of the variant 1 splice form of TIA-1 [86] was subcloned into pEYFP.C1 (Clonetech) yielding TIA-1-eYFP, (plasmid was courtesy of Dr. P. Anderson). In all constructs the fluorophor is N-terminal to the protein of interest.

### Cell Culture

DDT1-MF2 Hamster smooth muscle cells were grown in 100 mm dishes at 37°C, 5% CO_2 _in Dubelcco's Modified Eagle Medium (Invitrogen) supplemented with 5% Fetal Bovine Serum (Hyclone) and 0.1% penicillin/streptomycin solution (Invitrogen). For transient transfection DDT1-MF2 cells were grown on 25 mm glass cover slips to approximately 80% confluence. For immunocytochemistry DDT1-MF2 cells were grown on 18 mm glass cover slips to approximately 60% confluence. Where indicated, cells were treated with anisomycin (75 nM) or sodium arsenite (0.5 mM).

### Transient Transfection of Plasmid Vectors

Vectors were transfected into DDT1-MF2 using Fugene 6 (Roche) following the manufacturers protocol. The amount of plasmid DNA transfected was adjusted empirically to yield the desired 1:1 molar ratio of the CFP to YFP protein.

### Immunocytochemistry

DDT1-MF2 cells were fixed with 4% paraformaldehyde (Electron Microscopy Sciences), permeabilized with 0.5% Triton X-100 in PHEM buffer (60 mM PIPES, 25 mM Hepes, 10 mM EGTA, 2 mM MgCl_2_, pH 6.9), treated with 0.1% sodium borohydride (Sigma) and blocked with 5% horse serum. Cells were incubated at 4°C, overnight with 1°Ab and in 2°Ab for 1 hour at room temperature in the dark. Cover slips were mounted on glass slides using a 12.5% solution of Mowiol (Calbiochem) in 50% glycerol. Detection of HuR was via a 1:400 dilution of monoclonal 1°Ab (Santa Cruz Biotechnology) or a 1:200 dilution of a rabbit polyclonal 1°Ab; detection of AUF1 was via a 1: 500 dilution of a polyclonal 1°Ab (Upstate) which recognizes all 4 isoforms of AUF1; detection of TIA-1 was via a 1:500 dilution of goat polyclonal 1°Ab (Santa Cruz Biotechnology); detection of KSRP was via a 1:1000 dilution of a mouse monoclonal 1°Ab (courtesy of Dr. D.L. Black) and detection of Hedls was via a 1:8000 dilution of a rabbit polyclonal 1°Ab (courtesy of Dr. J. Lykke-Anderson). Secondary antibodies used were either a 1:200 dilution of goat anti-rabbit 2°Ab labeled with Cy5; a 1:400 dilution of a goat anti-rabbit 2°Ab labeled with Cy3; a 1:800 dilution of goat anti-mouse 2°Ab labeled with Cy5; a 1:1000 dilution of goat anti-mouse 2°Ab labeled with Cy3; a 1:2000 dilution of goat anti-mouse 2° Ab labeled with Alexa Fluor 488; a 1:200 dilution of a donkey anti-goat Cy3 or a 1:400 dilution of a donkey anti-goat 2°Ab labeled with Alexa Fluor 647. With the exception of TIA-1, all 1° and 2° Abs were applied together. To prevent cross-reaction between 2°Ab, the TIA-1 donkey anti-goat 2°Ab was added first and separately. All Cy3 and Cy5 labeled 2° antibodies were F(ab')_2 _IgG fragments from Jackson Immuno Research; Alexa Fluor dye labeled antibodies were F(ab')_2 _IgG fragments from Molecular Probes.

### Immuno-FRET

For detection of a FRET signal via labeled 2° Ab, DDT1-MF2 cells were processed for immunocytochemistry as indicated above. The fluorophors Cy3 and Cy5 or Alexa Fluor 647 (Invitrogen) conjugated to 2° Ab were used. In this instance Cy3 is the donor fluorophor and Cy5 or Alexa Fluor 647 is the acceptor fluorophor. In DDT1-MF2 cells, the signal intensity ratio of Cy3 to Cy5 required for energy transfer to occur and generate a FRET signal was determined to be 1:1 or greater (data not shown). The requirements for FRET; a FRET fluorophor pair, specific distance between fluorophors and fluorophor alignment, are not changed due to placing the fluorophor pair on 2° Ab.

### Microscopy

Images of live and fixed cells were captured at 100× magnification under oil immersion, (lens numerical aperture 1.40), using 2 × 2 or 4 × 4 binning mode on an inverted Nikon Eclipse TE3000 fluorescence microscope using a Cooke SensiCam QE CCD camera running Slidebook software (3I, version 4.0.1.43). All images were collected at room temperature using an integration time greater than 25 ms. Additionally, all images were background corrected by subtracting the intensity of a cell "devoid" area from each individual image. For live cell images, imaging medium was DMEM and integration time was between 25 and 250 ms. For fixed cell and immunostained images, integration time was between 25 and 1000 ms. Live cells containing CFP and YFP labeled proteins were viewed via the CFP channel to prevent photobleaching of YFP. All images presented had a signal intensity greater than 100 after background correction. FRETc images were produced using Slidebook software (3I, version 4.0.1.43) using spectral bleed through numbers that had been experimentally determined as detailed below. Images were edited using Adobe Illustrator.

All experimental data was collected from a minimum of two separate experiments. For each immunocytochemical experiment, a minimum of 400 cells was examined. For each transfection experiment, a minimum of 200 cells was examined. In general, images were captured at a level that would provide best resolution of the nucleus.

### Spectral bleed through calculations

The CFP and YFP spectral bleed through numbers were calculated as indicated previously [15]. Additionally, the spectral bleed through number of TIA-1-eYFP vector was determined. As a further proof of the bleed through numbers, and to show that the mere cellular presence of FRET pair fluorophors does not generate a FRET signal, the transient transfection of TIA-1-eYFP and the eCFP vector was carried out to add to the controls previously conducted for empirical correction of the spectral bleed through number [15]. The Cy3 and Cy5 spectral bleed through numbers were determined by imaging KSRP, AUF1, Dcp1a, Hedls, HuR and TIA-1 with Cy3-labelled 2°Ab; KSRP, AUF1, Dcp1a, Hedls and HuR with Cy5-labelled 2°Ab and TIA-1 with Alexa Fluor 647-labeled 2°Ab. The spectral bleed through number was also determined for the Cy3-labeled β-AR mRNA. As a further proof of the bleed through numbers, and a control for the FRET signal generation immunostaining of non interacting proteins with the FRET pair, Cy3-Cy5, was used for empirical correction of the spectral bleed through number; Cy5 labeled 2°Ab detection of KSRP, AUF1, Hedls with Cy3 labeled anti β-tubulin 1°Ab (Sigma) and Alexa Fluor 647-labelled 2°Ab detection of TIA-1 with Cy3 labeled anti-β-tubulin 1°Ab (Sigma). A similar control was conducted for the Cy3-labeled mRNA with transiently transfected TTP-GFP, AUF1-GFP and the GFP vector. FRET positive control were conducted using Cy3 and Cy5 2°Ab staining to a single 1°Ab recognizing KSRP, AUF1, Dcp1a, Hedls, HuR and TIA-1.

### Calculation of FRETc

To calculate FRETc, the following equation was used:

FRETc=raw FRET−(D*X)−(A*Y)

FRETc is the corrected FRET value, raw FRET is the uncorrected FRET intensity, D is the donor fluorophor intensity, X is the donor fluorophor spectral bleed through number, A is the acceptor fluorophor intensity, and Y is the acceptor fluorophor spectral bleed through number.

### *In vitro *transcription of Cy3-labeled mRNAs

To create ribonuclease resistant RNA labeled with a fluorescent Cy3 moiety, we incorporated Cy3-UTP (Amersham) and 2'F substituted CTP and UTP (Epicenter Biotechnologies) into β1 Adrenergic Receptor mRNA molecules via T7 RNA polymerase (Epicenter Technologies). We altered previously published methods [87], to incorporate a 5' m^7^G cap analogue (Promega) and 3' polyadenylation to the stabilized, fluorescent RNA molecule. A DNA template (1 μg) with an incorporated T7 promoter sequence was transcribed using Durascribe T7 enzyme (2 μl) in a 20 μl reaction containing 5 mM ATP, 1 mM GTP, 5 mM 2'F-dCTP, 5 mM 2'F-dUTP, 0.75 mM Cy3-UTP, 10 mM DTT and 4 mM m^7^G cap analogue for 2 hours at 37°C. Polyadenylation was carried out subsequent to the *in vitro* transcription. After removal of a small aliquot (1 μl) for gel electrophoresis, 3.125 mM MnCl_2_, 1.25 mM ATP, 2 ul E-PAP enzyme, 10 ul 5× E-PAP buffer and 1 μl SUPERase, (Ambion) were added directly to the *in vitro *transcription reaction tube, total volume 40 μl, incubate 2 hours at 37°C. This reaction creates a poly A tail of ~100-200 nucleotides. Based on a calculation of the base:dye ratio (i.e., UTP to Cy3-UTP ratio), the capped and polyadenylated Cy3-RNA products contain ~2-4 Cy3 molecules per transcript. The optimal concentration for observation of diffuse Cy3 signal in cells has been determined empirically to be ~200 pM of RNA when DDT1-MF2 cells were transfected using Lipofectamine 2000, as per the manufacturer's guidelines (Invitrogen).

### Transient Transfection of Cy3-labeled RNA

Cy3-labeled RNAs were transfected into DDT1-MF2 cells using Lipofectamine 2000 (Invitrogen). Briefly, DDT1-MF2 cells were resuspended and plated at 80% confluence onto 100 mm plates containing 25 mm cover slips and allowed to adhere, about 2 hours. The RNA was melted at 90°C for 1 min and allowed to cool slowly to room temperature prior to transfection. The Lipofectamine 2000 and Cy3-labeled RNA (2 μg) were diluted separately into Optimem 1 media (4 fold and 140 fold respectively) (Invitrogen) and incubated at room temperature for 30 minutes. DDT1-MF2 cells were washed with Optimem 1 and changed into this media. The Lipofectamine 2000 and RNA solutions were mixed together and added to the media bathing the cells. The cells were incubated overnight at 37°C, 5% CO_2 _prior to the media being changed back to DMEM (37°C). Cells were left to recover for 4 hours prior to any subsequent treatment.

## Authors' contributions

PDG performed all FRET and immuno-FRET experiments between RNA binding proteins, performed all the image analysis, created all the figures, and wrote various drafts of the manuscript. MAT performed all FRET experiments between fluorescently labeled RNA and RNA binding proteins, created preliminary figures, and assisted in writing the methods section relevant to these experiments. JDP is the laboratory director, and oversaw all aspects of the project. JDP worked with PDG to design and analyze the data, and wrote major portions of the manuscript as well as performing all final editing. All authors approve of the final manuscript.

## Author's information

**PDG **is a post-doctoral fellow in the JDP lab and has been working in the area of RNA binding protein imaging for several years. **MAT **is a graduate student in pharmacology, currently working in the area of cancer biology. **JDP **has been a Professor of Medicine and Pharmacology at the University of Colorado School of Medicine since 1991. His laboratory has been focused on RNA turnover and signaling pathways that modulate the function of RNA binding proteins for many years. JDP has co-organized and participated in numerous meetings related to RNA turnover and RNA biology.

## Supplementary Material

Additional file 1 **Video 1**. Interactions of PBs and SGs in DDT1-MF2 cells subjected to oxidative stress. DDT1-MF2 cells were treated with 0.5 mM sodium arsenite for 30 minutes prior to immunostaining. PBs were detected by Hedls (red) and SGs by KSRP (green). Images were collected along the Z axis of the cell at 100× magnification under oil immersion, (lens numerical aperture 1.40), using 2 × 2 binning mode on an inverted Nikon Eclipse TE3000 fluorescence microscope using a Cooke SensiCam QE CCD camera running Slidebook software (3I, version 4.0.1.43). Capture time was 500 ms and step size was 0.5 microns. 3D stack was "deconvolved" using the nearest-neighbors method. Click here for file

## References

[B1] Ross J mRNA stability in mammalian cells Microbiological Reviews 1995 59 423 450 7565413 10.1128/mr.59.3.423-450.1995 PMC239368

[B2] Zhang W Wagner BJ Ehrenman K Schaefer AW DeMaria CT Crater D DeHaven K Long L Brewer G Purification, characterization, and cDNA cloning of an AU-rich element RNA-binding protein, AUF1 Mol Cell Biol 1993 13 12 7652 7665 8246982 10.1128/mcb.13.12.7652-7665.1993 PMC364837

[B3] Stoecklin G Colombi M Raineri I Leuenberger S Mallaun M Schmidlin M Gross B Lu M Kitamura T Moroni C Functional cloning of BRF1, a regulator of ARE-dependent mRNA turnover EMBO J 2002 21 17 4709 4718 10.1093/emboj/cdf444 12198173 PMC126184

[B4] Ma W-J Cheng S Campbell C Wright A Furneaux H Cloning and characterization of HuR, a ubiquitously expressed elav-like protein J Biol Chem 1996 271 8144 8151 10.1074/jbc.271.14.8144 8626503

[B5] Min H Turck CW Nikolic JM Black DL A new regulatory protein, KSRP, mediates exon inclusion through an intronic splicing enhancer Genes Dev 1997 11 8 1023 1036 10.1101/gad.11.8.1023 9136930

[B6] Anderson P TIA-1: structural and functional studies on a new class of cytolytic effector molecule Curr Top Microbiol Immunol 1995 198 131 143 7774278 10.1007/978-3-642-79414-8_8

[B7] Lai WS Carballo E Strum JR Kennington EA Phillips RS Blackshear PJ Evidence that tristetraprolin binds to AU-rich elements and promotes the deadenylation and destabilization of tumor necrosis factor alpha mRNA Mol Cell Biol 1999 19 6 4311 4323 10330172 10.1128/mcb.19.6.4311 PMC104391

[B8] Lai WS Thompson MJ Blackshear PJ Characteristics of the intron involvement in the mitogen-induced expression of Zfp-36 J Biol Chem 1998 273 1 506 517 10.1074/jbc.273.1.506 9417109

[B9] Wagner BJ DeMaria CT Sun Y Wilson GM Brewer G Structure and genomic organization of the human AUF1 gene: alternative pre-mRNA splicing generates four protein isoforms Genomics 1998 48 2 195 202 10.1006/geno.1997.5142 9521873

[B10] Lal A Mazan-Mamczarz K Kawai T Yang X Martindale JL Gorospe M Concurrent versus individual binding of HuR and AUF1 to common labile target mRNAs EMBO J 2004 23 3092 3102 10.1038/sj.emboj.7600305 15257295 PMC514922

[B11] Izquierdo JM Control of the ATP synthase beta subunit expression by RNA-binding proteins TIA-1, TIAR, and HuR Biochem Biophys Res Commun 2006 348 2 703 711 10.1016/j.bbrc.2006.07.114 16890199

[B12] Kawai T Lal A Yang X Galban S Mazan-Mamczarz K Gorospe M Translational control of cytochrome c by RNA-binding proteins TIA-1 and HuR Mol Cell Biol 2006 26 8 3295 3307 10.1128/MCB.26.8.3295-3307.2006 16581801 PMC1446930

[B13] Ruggiero T Trabucchi M Ponassi M Corte G Chen CY al-Haj L Khabar KS Briata P Gherzi R Identification of a set of KSRP target transcripts upregulated by PI3K-AKT signaling BMC Molecular Biology 2007 8 28 10.1186/1471-2199-8-28 17437629 PMC1858702

[B14] Rothe F Gueydan C Bellefroid E Huez G Kruys V Identification of FUSE-binding proteins as interacting partners of TIA proteins Biochem Biophys Res Commun 2006 343 1 57 68 10.1016/j.bbrc.2006.02.112 16527256

[B15] David PS Tanveer R Port JD FRET-detectable interactions between the ARE binding proteins, HuR and p37AUF1 RNA 2007 13 9 1453 1468 10.1261/rna.501707 17626845 PMC1950754

[B16] Palanisamy V Park NJ Wang J Wong DT AUF1 and HuR proteins stabilize interleukin-8 mRNA in human saliva J Dent Res 2008 87 8 772 776 10.1177/154405910808700803 18650551 PMC2572714

[B17] Pan YX Chen H Kilberg MS Interaction of RNA-binding proteins HuR and AUF1 with the human ATF3 mRNA 3'-untranslated region regulates its amino acid limitation-induced stabilization J Biol Chem 2005 280 41 34609 34616 10.1074/jbc.M507802200 16109718 PMC3600371

[B18] Suswam EA Nabors LB Huang Y Yang X King PH IL-1beta induces stabilization of IL-8 mRNA in malignant breast cancer cells via the 3' untranslated region: Involvement of divergent RNA-binding factors HuR, KSRP and TIAR Int J Cancer 2005 113 6 911 919 10.1002/ijc.20675 15514971

[B19] Chen CY Gherzi R Ong SE Chan EL Raijmakers R Pruijn GJ Stoecklin G Moroni C Mann M Karin M AU binding proteins recruit the exosome to degrade ARE-containing mRNAs Cell 2001 107 4 451 464 10.1016/S0092-8674(01)00578-5 11719186

[B20] Linker K Pautz A Fechir M Hubrich T Greeve J Kleinert H Involvement of KSRP in the post-transcriptional regulation of human iNOS expression-complex interplay of KSRP with TTP and HuR Nucleic Acids Res 2005 33 15 4813 4827 10.1093/nar/gki797 16126846 PMC1192834

[B21] Nechama M Ben-Dov IZ Briata P Gherzi R Naveh-Many T The mRNA decay promoting factor K-homology splicing regulator protein post-transcriptionally determines parathyroid hormone mRNA levels FASEB Journal 2008 22 10 3458 3468 10.1096/fj.08-107250 18583400

[B22] Cok SJ Acton SJ Sexton AE Morrison AR Identification of RNA-binding Proteins in RAW 264.7 Cells That Recognize a Lipopolysaccharide-responsive Element in the 3-Untranslated Region of the Murine Cyclooxygenase-2 mRNA J Biol Chem 2004 279 9 8196 8205 10.1074/jbc.M308475200 14662769

[B23] Pullmann R Jr Kim HH Abdelmohsen K Lal A Martindale JL Yang X Gorospe M Analysis of turnover and translation regulatory RNA-binding protein expression through binding to cognate mRNAs Mol Cell Biol 2007 27 18 6265 6278 10.1128/MCB.00500-07 17620417 PMC2099612

[B24] Zhu H Hinman MN Hasman RA Mehta P Lou H Regulation of neuron-specific alternative splicing of neurofibromatosis type 1 pre-mRNA Mol Cell Biol 2008 28 4 1240 1251 10.1128/MCB.01509-07 18086893 PMC2258745

[B25] Doller A Schlepckow K Schwalbe H Pfeilschifter J Eberhardt W Tandem phosphorylation of serines 221 and 318 by protein kinase Cdelta coordinates mRNA binding and nucleocytoplasmic shuttling of HuR Mol Cell Biol 2010 30 6 1397 1410 10.1128/MCB.01373-09 20086103 PMC2832487

[B26] Diaz-Moreno I Hollingworth D Frenkiel TA Kelly G Martin S Howell S Garcia-Mayoral M Gherzi R Briata P Ramos A Phosphorylation-mediated unfolding of a KH domain regulates KSRP localization via 14-3-3 binding Nat Struct Mol Biol 2009 16 3 238 246 10.1038/nsmb.1558 19198587 PMC2858377

[B27] Wilson GM Lu J Sutphen K Sun Y Huynh Y Brewer G Regulation of A + U-rich Element-directed mRNA Turnover Involving Reversible Phosphorylation of AUF1 J Biol Chem 2003 278 35 33029 33038 10.1074/jbc.M305772200 12819195

[B28] Sandler H Stoecklin G Control of mRNA decay by phosphorylation of tristetraprolin Biochem Soc Trans 2008 36 Pt 3 491 496 10.1042/BST0360491 18481987

[B29] Maitra S Chou CF Luber CA Lee KY Mann M Chen CY The AU-rich element mRNA decay-promoting activity of BRF1 is regulated by mitogen-activated protein kinase-activated protein kinase 2 Rna 2008 14 5 950 959 10.1261/rna.983708 18326031 PMC2327367

[B30] Abdelmohsen K Kim MM Srikantan S Mercken EM Brennan SE Wilson GM de Cabo R Gorospe M miR-519 suppresses tumor growth by reducing HuR levels Cell Cycle 2010 9 7 1354 1359 10.4161/cc.9.7.11164 PMC3057889 20305372

[B31] Konig P Krasteva G Tag C Konig IR Arens C Kummer W FRET-CLSM and double-labeling indirect immunofluorescence to detect close association of proteins in tissue sections Lab Invest 2006 86 8 853 864 16783395 10.1038/labinvest.3700443

[B32] Kulkarni M Ozgur S Stoecklin G On track with P-bodies Biochem Soc Trans 2010 38 Pt 1 242 251 10.1042/BST0380242 20074068

[B33] Fenger-Gron M Fillman C Norrild B Lykke-Andersen J Multiple processing body factors and the ARE binding protein TTP activate mRNA decapping Mol Cell 2005 20 6 905 915 10.1016/j.molcel.2005.10.031 16364915

[B34] Ingelfinger D Arndt-Jovin DJ Luhrmann R Achsel T The human LSm1-7 proteins colocalize with the mRNA-degrading enzymes Dcp1/2 and Xrnl in distinct cytoplasmic foci RNA 2002 8 12 1489 1501 12515382 PMC1370355

[B35] Fernandez-Suarez M Baruah H Martinez-Hernandez L Xie KT Baskin JM Bertozzi CR Ting AY Redirecting lipoic acid ligase for cell surface protein labeling with small-molecule probes Nat Biotechnol 2007 25 12 1483 1487 10.1038/nbt1355 18059260 PMC2654346

[B36] Gherzi R Trabucchi M Ponassi M Ruggiero T Corte G Moroni C Chen CY Khabar KS Andersen JS Briata P The RNA-binding protein KSRP promotes decay of beta-catenin mRNA and is inactivated by PI3K-AKT signaling Plos Biology 2006 5 1 e5 10.1371/journal.pbio.0050005 17177604 PMC1702562

[B37] Gilks N Kedersha N Ayodele M Shen L Stoecklin G Dember LM Anderson P Stress granule assembly is mediated by prion-like aggregation of TIA-1 Mol Biol Cell 2004 15 12 5383 5398 10.1091/mbc.E04-08-0715 15371533 PMC532018

[B38] Kedersha N Cho MR Li W Yacono PW Chen S Gilks N Golan DE Anderson P Dynamic shuttling of TIA-1 accompanies the recruitment of mRNA to mammalian stress granules J Cell Biol 2000 151 6 1257 1268 10.1083/jcb.151.6.1257 11121440 PMC2190599

[B39] Kedersha NL Gupta M Li W Miller I Anderson P RNA-binding proteins TIA-1 and TIAR link the phosphorylation of eIF-2 alpha to the assembly of mammalian stress granules J Cell Biol 1999 147 7 1431 1442 10.1083/jcb.147.7.1431 10613902 PMC2174242

[B40] Anderson P Kedersha N Stress granules: the Tao of RNA triage Trends Biochem Sci 2008 33 3 141 150 18291657 10.1016/j.tibs.2007.12.003

[B41] Anderson P Kedersha N Stress granules Curr Biol 2009 19 10 R397 398 10.1016/j.cub.2009.03.013 19467203

[B42] Kedersha N Anderson P Stress granules: sites of mRNA triage that regulate mRNA stability and translatability Biochem Soc Trans 2002 30 Pt 6 963 969 12440955 10.1042/bst0300963

[B43] Kedersha N Stoecklin G Ayodele M Yacono P Lykke-Andersen J Fitzler MJ Scheuner D Kaufman RJ Golan DE Anderson P Stress granules and processing bodies are dynamically linked sites of mRNP remodeling J Cell Biol 2005 169 6 871 884 10.1083/jcb.200502088 15967811 PMC2171635

[B44] Newbury SF Muhlemann O Stoecklin G Turnover in the Alps: an mRNA perspective. Workshops on mechanisms and regulation of mRNA turnover EMBO Rep 2006 7 2 143 148 10.1038/sj.embor.7400628 16439997 PMC1369256

[B45] Kedersha N Tisdale S Hickman T Anderson P Real-time and quantitative imaging of mammalian stress granules and processing bodies Methods Enzymol 2008 448 521 552 full_text 19111193 10.1016/S0076-6879(08)02626-8

[B46] Gallouzi IE Brennan CM Stenberg MG Swanson MS Eversole A Maizels N Steitz JA HuR binding to cytoplasmic mRNA is perturbed by heat shock Proc Natl Acad Sci USA 2000 97 7 3073 3078 10.1073/pnas.97.7.3073 10737787 PMC16194

[B47] Eulalio A Rehwinkel J Stricker M Huntzinger E Yang SF Doerks T Dorner S Bork P Boutros M Izaurralde E Target-specific requirements for enhancers of decapping in miRNA-mediated gene silencing Genes Dev 2007 21 20 2558 2570 10.1101/gad.443107 17901217 PMC2000321

[B48] Parker R Sheth U P bodies and the control of mRNA translation and degradation Mol Cell 2007 25 5 635 646 10.1016/j.molcel.2007.02.011 17349952

[B49] Anderson P Kedersha N RNA granules: post-transcriptional and epigenetic modulators of gene expression Nat Rev Mol Cell Biol 2009 10 6 430 436 10.1038/nrm2694 19461665

[B50] Franks TM Lykke-Andersen J The control of mRNA decapping and P-body formation Mol Cell 2008 32 5 605 615 10.1016/j.molcel.2008.11.001 19061636 PMC2630519

[B51] Pedro-Segura E Vergara SV Rodriguez-Navarro S Parker R Thiele DJ Puig S The Cth2 ARE-binding protein recruits the Dhh1 helicase to promote the decay of succinate dehydrogenase SDH4 mRNA in response to iron deficiency J Biol Chem 2008 283 42 28527 28535 10.1074/jbc.M804910200 18715869 PMC2568921

[B52] Headley VV Tanveer R Greene SM Zweifach A Port JD Reciprocal regulation of beta-adrenergic receptor mRNA stability by mitogen activated protein kinase activation and inhibition Mol Cell Biochem 2004 258 1-2 109 119 10.1023/B:MCBI.0000012841.03400.42 15030175

[B53] Kontoyiannis D Kotlyarov A Carballo E Alexopoulou L Blackshear PJ Gaestel M Davis R Flavell R Kollias G Interleukin-10 targets p38 MAPK to modulate ARE-dependent TNF mRNA translation and limit intestinal pathology Embo J 2001 20 14 3760 3770 10.1093/emboj/20.14.3760 11447117 PMC125555

[B54] Ming XF Stoecklin G Lu M Looser R Moroni C Parallel and independent regulation of interleukin-3 mRNA turnover by phosphatidylinositol 3-kinase and p38 mitogen-activated protein kinase Mol Cell Biol 2001 21 17 5778 5789 10.1128/MCB.21.17.5778-5789.2001 11486017 PMC87297

[B55] Clark AR Dean JL Saklatvala J The p38 MAPK pathway mediates both antiinflammatory and proinflammatory processes: comment on the article by Damjanov and the editorial by Genovese Arthritis Rheum 2009 60 11 3513 3514 10.1002/art.24919 19877029

[B56] Kedersha N Anderson P Mammalian stress granules and processing bodies Methods Enzymol 2007 431 61 81 full_text 17923231 10.1016/S0076-6879(07)31005-7

[B57] Singer RH RNA localization: visualization in real-time Curr Biol 2003 13 17 R673 675 10.1016/S0960-9822(03)00605-5 12956970

[B58] Anderson P Kedersha N RNA granules J Cell Biol 2006 172 6 803 808 10.1083/jcb.200512082 16520386 PMC2063724

[B59] Panyi G Bagdany M Bodnar A Vamosi G Szentesi G Jenei A Matyus L Varga S Waldmann TA Gaspar R Damjanovich S Colocalization and nonrandom distribution of Kv1.3 potassium channels and CD3 molecules in the plasma membrane of human T lymphocytes Proc Natl Acad Sci USA 2003 100 5 2592 2597 10.1073/pnas.0438057100 12604782 PMC151385

[B60] Krasteva G Pfeil U Drab M Kummer W Konig P Caveolin-1 and -2 in airway epithelium: expression and in situ association as detected by FRET-CLSM Respir Res 2006 7 108 10.1186/1465-9921-7-108 16904002 PMC1563466

[B61] Cayli S Klug J Chapiro J Frohlich S Krasteva G Orel L Meinhardt A COP9 signalosome interacts ATP-dependently with p97/valosin-containing protein (VCP) and controls the ubiquitination status of proteins bound to p97/VCP J Biol Chem 2009 284 50 34944 34953 10.1074/jbc.M109.037952 19826004 PMC2787357

[B62] Cerecedo D Cisneros B Suarez-Sanchez R Hernandez-Gonzalez E Galvan I beta-Dystroglycan modulates the interplay between actin and microtubules in human-adhered platelets Br J Haematol 2008 141 4 517 528 10.1111/j.1365-2141.2008.07048.x 18341635

[B63] Wilson GM Sun Y Lu H Brewer G Assembly of AUF1 oligomers on U-rich RNA targets by sequential dimer association J Biol Chem 1999 274 47 33374 33381 10.1074/jbc.274.47.33374 10559216

[B64] DeMaria CT Brewer G AUF1 binding affinity to A+U-rich elements correlates with rapid mRNA degradation J Biol Chem 1996 271 21 12179 12184 10.1074/jbc.271.21.12179 8647811

[B65] DeMaria CT Sun Y Long L Wagner BJ Brewer G Structural determinants in AUF1 required for high affinity binding to A + U-rich elements J Biol Chem 1997 272 44 27635 27643 10.1074/jbc.272.44.27635 9346902

[B66] Balagopal V Parker R Polysomes, P bodies and stress granules: states and fates of eukaryotic mRNAs Curr Opin Cell Biol 2009 21 3 403 408 10.1016/j.ceb.2009.03.005 19394210 PMC2740377

[B67] Buchan JR Parker R Eukaryotic stress granules: the ins and outs of translation Mol Cell 2009 36 6 932 941 10.1016/j.molcel.2009.11.020 20064460 PMC2813218

[B68] Briata P Forcales SV Ponassi M Corte G Chen CY Karin M Puri PL Gherzi R p38-dependent phosphorylation of the mRNA decay-promoting factor KSRP controls the stability of select myogenic transcripts Mol Cell 2005 20 6 891 903 10.1016/j.molcel.2005.10.021 16364914

[B69] Doller A el Akool S Huwiler A Muller R Radeke HH Pfeilschifter J Eberhardt W Posttranslational modification of the AU-rich element binding protein HuR by protein kinase Cdelta elicits angiotensin II-induced stabilization and nuclear export of cyclooxygenase 2 mRNA Mol Cell Biol 2008 28 8 2608 2625 10.1128/MCB.01530-07 18285462 PMC2293108

[B70] Doller A Huwiler A Muller R Radeke HH Pfeilschifter J Eberhardt W Protein kinase C alpha-dependent phosphorylation of the mRNA-stabilizing factor HuR: implications for posttranscriptional regulation of cyclooxygenase-2 Mol Biol Cell 2007 18 6 2137 2148 10.1091/mbc.E06-09-0850 17392515 PMC1877114

[B71] Doller A Pfeilschifter J Eberhardt W Signalling pathways regulating nucleo-cytoplasmic shuttling of the mRNA-binding protein HuR Cell Signal 2008 20 2165 2173 10.1016/j.cellsig.2008.05.007 18585896

[B72] Kim HH Gorospe M Phosphorylated HuR shuttles in cycles Cell Cycle 2008 7 20 3124 3126 18927508 10.4161/cc.7.20.6884 PMC2577782

[B73] Kim HH Yang X Kuwano Y Gorospe M Modification at HuR(S242) alters HuR localization and proliferative influence Cell Cycle 2008 7 21 3371 3377 18948743 10.4161/cc.7.21.6895 PMC2704553

[B74] Kim HH Abdelmohsen K Lal A Pullmann R Jr Yang X Galban S Srikantan S Martindale JL Blethrow J Shokat KM Gorospe M Nuclear HuR accumulation through phosphorylation by Cdk1 Genes Dev 2008 22 13 1804 1815 10.1101/gad.1645808 18593881 PMC2492667

[B75] Abdelmohsen K Pullmann R Jr Lal A Kim HH Galban S Yang X Blethrow JD Walker M Shubert J Gillespie DA Furneaux H Gorospe M Phosphorylation of HuR by Chk2 regulates SIRT1 expression Mol Cell 2007 25 4 543 557 10.1016/j.molcel.2007.01.011 17317627 PMC1986740

[B76] Li H Park S Kilburn B Jelinek MA Henschen-Edman A Aswad DW Stallcup MR Laird-Offringa IA Lipopolysaccharide-induced methylation of HuR, an mRNA-stabilizing protein, by CARM1. Coactivator-associated arginine methyltransferase J Biol Chem 2002 277 47 44623 44630 10.1074/jbc.M206187200 12237300

[B77] Sun L Stoecklin G Van Way S Hinkovska-Galcheva V Guo RF Anderson P Shanley TP Tristetraprolin (TTP)-14-3-3 complex formation protects TTP from dephosphorylation by protein phosphatase 2a and stabilizes tumor necrosis factor-alpha mRNA J Biol Chem 2007 282 6 3766 3777 10.1074/jbc.M607347200 17170118

[B78] He C Schneider R 14-3-3sigma is a p37 AUF1-binding protein that facilitates AUF1 transport and AU-rich mRNA decay Embo J 2006 25 16 3823 3831 10.1038/sj.emboj.7601264 16902409 PMC1553187

[B79] Schmidlin M Lu M Leuenberger SA Stoecklin G Mallaun M Gross B Gherzi R Hess D Hemmings BA Moroni C The ARE-dependent mRNA-destabilizing activity of BRF1 is regulated by protein kinase B Embo J 2004 23 24 4760 4769 10.1038/sj.emboj.7600477 15538381 PMC535089

[B80] Morrison DK The 14-3-3 proteins: integrators of diverse signaling cues that impact cell fate and cancer development Trends Cell Biol 2009 19 1 16 23 10.1016/j.tcb.2008.10.003 19027299 PMC3073487

[B81] Barreau C Dutertre S Paillard L Osborne HB Liposome-mediated RNA transfection should be used with caution RNA 2006 12 10 1790 1793 10.1261/rna.191706 16921069 PMC1581979

[B82] Wilkie GS Davis I Drosophila wingless and pair-rule transcripts localize apically by dynein-mediated transport of RNA particles Cell 2001 105 2 209 219 10.1016/S0092-8674(01)00312-9 11336671

[B83] Lorenz M Visualizing protein-Rna interactions inside cells by fluorescence resonance energy transfer Rna 2009 15 1 97 103 10.1261/rna.1307809 19033374 PMC2612761

[B84] Huranova M Jablonski JA Benda A Hof M Stanek D Caputi M In vivo detection of Rna-binding protein interactions with cognate RNA sequences by fluorescence resonance energy transfer Rna 2009 15 11 2063 2071 10.1261/rna.1678209 19767419 PMC2764471

[B85] Galperin E Verkhusha VV Sorkin A Three-chromophore FRET microscopy to analyze multiprotein interactions in living cells Nat Methods 2004 1 3 209 217 10.1038/nmeth720 15782196

[B86] Kawakami A Tian Q Streuli M Poe M Edelhoff S Disteche CM Anderson P Intron-exon organization and chromosomal localization of the human TIA-1 gene J Immunol 1994 152 10 4937 4945 8176212

[B87] Capodici J Kariko K Weissman D Inhibition of HIV-1 infection by small interfering RNA-mediated RNA interference J Immunol 2002 169 9 5196 5201 12391237 10.4049/jimmunol.169.9.5196

